# Temporal scale and conspecific density drive moonlight preferences of a nocturnal granivore

**DOI:** 10.1093/beheco/araf150

**Published:** 2026-02-27

**Authors:** Rafał Zwolak, Milena Zduniak, Paulina Celebias, Aleksandra Wróbel

**Affiliations:** Department of Systematic Zoology, Adam Mickiewicz University, Uniwersytetu Poznańskiego 6, 61-614 Poznań, Poland; Polish Academy of Sciences, Mammal Research Institute, Stoczek 1, 17-230 Białowieża, Poland; Department of Systematic Zoology, Adam Mickiewicz University, Uniwersytetu Poznańskiego 6, 61-614 Poznań, Poland; Polish Academy of Sciences, Mammal Research Institute, Stoczek 1, 17-230 Białowieża, Poland

**Keywords:** density dependence, individual traits, intraspecific variation, risk allocation hypothesis, foraging strategies, visual acuity hypothesis

## Abstract

Foraging animals must balance energy gain with predation risk. In nocturnal animals, this tradeoff is often mediated by moonlight, which helps to find food but also increases exposure to predators. Although most studies suggest rodents avoid moonlight, prior research has largely focused on open habitats and used lunar phase as a crude proxy for illumination. We investigated how fine-scale moonlight brightness affects foraging in yellow-necked mice (*Apodemus flavicollis*) removing *Quercus robur* acorns in a broadleaf forest. Using complementary analyses, we examined foraging across nights and the timing of foraging bouts within nights. We expected that foraging would decrease with increasing moonlight, temporal preferences would be less pronounced under high mouse density, and sensitivity to moonlight would vary with residual reproductive value (influenced by sex and body mass) and behavioral type (exploration in open-field tests). Contrary to predictions, seed removal was higher during bright, clear nights. However, within nights, mice concentrated foraging efforts during darker intervals. This pattern is consistent with higher foraging efficiency on bright nights combined with avoidance of high-risk periods within those nights. As predicted, high mouse abundance reduced selectivity for optimal foraging conditions. While behavioral types had no effect, across-nights analyses demonstrated that males and lighter individuals preferred darker nights, while within nights, females showed greater sensitivity to cloud cover, foraging under lower moonlight when skies were clear. Overall, these results indicate that moonlight preferences can vary with the temporal scale of analyses and individual traits, while preference strength declines with increasing conspecific density.

## Teaser

Mice forage more during bright nights but select darker intervals within nights, balancing food gains against predation risk. Animals must find food without becoming food. Moonlight helps nocturnal mice find seeds but also exposes them to predators. We found that mice remove more seeds on bright nights but concentrate activity in darker periods within those nights. These decisions depend on moonlight intensity, mouse abundance, sex, and weight, showing how animals adjust their foraging activity to environmental risks.

## Introduction

Foraging animals must avoid being eaten. One key strategy is feeding when predators are less active or, more formally, allocating more feeding effort to low-risk periods and less to high-risk periods (Lima and Bednekoff 1999; Ferrari et al. 2009). However, the same environmental factors that increase the risk of predation can also make foragers more successful. Moonlight is a prime example: it can help nocturnal animals find food more easily, suggesting that bright nights might be optimal for feeding (the visual acuity hypothesis). On the other hand, it can expose foragers to predators, implying that animals should reduce feeding when moonlight is bright (the predation risk hypothesis) (Prugh and Golden 2014). Indeed, moonlight has long been studied in the context of foraging-predation tradeoffs (Kotler et al. 1991; Longland and Price 1991; Daly et al. 1992; Orrock et al. 2004). Most research shows that foragers avoid moonlight conditions, with exceptions such as visually oriented primates (Prugh and Golden 2014). Yet many of these studies have been conducted in open habitats, particularly deserts, under the assumption that moonlight can be ignored in denser environments like forests. That assumption, however, might be incorrect (Brivio et al. 2017; Gordigiani et al. 2022; Bischof et al. 2024) and merits further empirical evaluation.

Moreover, most studies used the moon phase as a crude proxy for average nighttime illumination, which poses several issues. First, the brightness of moonlight depends on more than just a lunar phase; cloud cover, season, latitude, and time of the night all play important roles (Śmielak 2023). Of these factors, only cloud cover has been considered relatively often (Prugh and Brashares 2010; Zaman et al. 2022; Cerri et al. 2023). Second, and more fundamentally, the temporal allocation of nocturnal animals' foraging activity involves 2 distinct processes that have rarely been separated (Śmielak 2023). The first is the selection of nights for foraging, which leads to between-nights variation in foraging activity. The second is the selection of specific times within each night to forage—or to avoid foraging—in order to reduce predation risk. This creates within-night variation in foraging activity. These processes are most relevant for animals with high metabolic rates and low energy reserves (eg, most small mammals) because such species cannot afford to skip foraging entirely on bright nights (Churchfield 2002; Hong-bi et al. 2020). Instead, they may shift feeding to darker periods within those nights. In such cases, the safer periods, though short, should show especially intense feeding effort (the risk allocation hypothesis: Lima and Bednekoff 1999). Focusing only on variation among nights ignores this pattern and can obscure behavioral responses to predation risk.

Another factor that has been understudied in the context of temporal variation in foraging activity is intraspecific abundance—even though abundance has been extensively examined in studies of habitat selection. According to habitat selection theory, when animals become abundant, they begin to occupy less-preferred habitats—a phenomenon captured by principle that “abundance destroys habitat selection” (Fretwell and Lucas 1970; Pulliam and Danielson 1991). This notion received strong empirical support (Petit and Petit 1996; Mobæk et al. 2009; Zwolak et al. 2016), and a similar process should apply to decisions *when* to forage. Specifically, animals may prefer certain periods, but when conspecific density is high, this preference can diminish. Thus, fluctuations in abundance can alter or mask animal responses to moonlight.

Finally, the influence of moonlight on foraging can vary according to individual traits. Animals with more proactive, highly exploratory behavioral types are expected to forage under riskier conditions than their more reactive counterparts, and thus should be less deterred by moonlight (Sih et al. 2004; Koolhaas et al. 2007; Toscano et al. 2016; Zwolak and Sih 2020). Additionally, males, who generally have greater potential reproductive rates (the maximum number of offspring possible) and can therefore benefit more from risk-taking (Glutton-Brock and Vincent 1991), are expected to be less cautious under high moonlight than females (Palanza 2001). Furthermore, risk-taking is affected by a tradeoff between current and future reproduction, often framed as the asset-protection principle (Clark 1994). However, the application of this principle varies depending on how “assets” are defined. If assets are interpreted as current reproductive investment (eg mates, territories, and offspring), then larger and older individuals have more to lose and are expected to be more risk-averse (Cresswell 1994). In contrast, if assets refer to future reproductive value (ie remaining reproductive potential), then smaller or younger individuals should behave more cautiously to preserve long-term fitness (Dammhahn 2012). In this case, such individuals would be expected to limit their activity under high moonlight, whereas larger or older ones would be relatively less responsive.

We explored these issues using the yellow-necked mouse (*Apodemus flavicollis*), a nocturnal granivore (Wójcik and Wołk 1985; Viviano et al. 2022) that forages on common oak (*Quercus robur*) acorns in a broadleaf forest. This species serves as a useful model system for studying seed removal and foraging behavior (Celebias et al. 2024; Mittelman et al. 2024; Zwolak et al. 2024b). We took advantage of masting-related fluctuations in mouse abundance to investigate changes in foraging activity over a wide gradient of conspecific density. Using individual-level data, we assessed the influence of mouse's traits on their foraging activity in relation to moonlight. We examined both between- and within-night variation in foraging to gain deeper insights into how individuals allocate time to this risky behavior. We used these complementary analyses to address 3 main questions: (i) How does moonlight affect the foraging of yellow-necked mice in a temperate broadleaf forest? (ii) How is the impact of moonlight modified by fluctuations in mouse abundance? (iii) How do individual traits of foraging mice alter their response to moonlight? We expected foraging activity to decline with increasing moonlight, both within and across nights, but anticipated that cloud cover would moderate this response. We also anticipated that the impact of moonlight would diminish with higher mouse abundance. Finally, we predicted that more exploratory mice, males, and heavier individuals would be less responsive to variation in moonlight and cloud cover than less exploratory mice, females, and lighter individuals.

## Methods

### Natural history

The yellow-necked mouse (15 to 60 g) is among the most widespread and abundant small rodents in European forests (Pucek 1984; Marsh et al. 2001). Although omnivorous, it obtains over 80% of its diet from tree seeds—primarily beech (*Fagus sylvatica*), hornbeam (*Carpinus betulus*), and oaks (*Q. robur* and *Q. petraea*; Selva et al. 2012). Populations of yellow-necked mice often increase in the year following a mast-seeding event but then typically crash due to low seed availability (Zwolak et al. 2016) and negative density dependence caused by reproductive suppression, predation, and parasitism intensifies (Jędrzejewski and Jędrzejewska 1993; Montgomery et al. 1997; Pedersen and Greives 2008).

Common oaks are prominent broad-leaved trees in European temperate woodlands. They hold significant economic value for timber trees and play a vital ecological role by providing habitats and resources for diverse animal communities (Mitchell et al. 2019). Their seed production is characterized by mast seeding, with synchronized yet highly variable crops across years. Acorns are large (1 to 6 g) and support a wide range of consumers, including insects (Bogdziewicz et al. 2018), rodents (Birkedal et al. 2009), birds (Bossema 1979; Wróbel et al. 2022), and ungulates (Touzot et al. 2023). Seed fall in common oaks occurs between September and November, peaking in October.

### Study sites and small mammal trapping

This research took place in the Zielonka Forest Landscape Park (52.6 N, 16.9 E), Greater Poland Voivodeship, Poland, during the summers of 2020 to 2022. The climate of this area is temperate, with mean temperatures ranging from −2.5 °C in January to 18.2 °C in July, and about 520 mm of annual precipitation. We selected 6 hardwood-dominated forest sites, characterized by stands of European beech (*F. sylvatica*) and oaks (*Q. robur, Q. petraea*), and accompanied by Scots pine (*Pinus sylvestris*) and European larch (*Larix decidua*). The stands were about 80 years old and managed for wood. Beech-dominated forests naturally have sparse understory, and in these managed stands, it is even sparser, with little downed wood and a homogeneous forest floor. Distances among sites ranged from 0.3 to 3.5 km (mean = 1.9 km), ensuring spatial independence while remaining within the same continuous forest habitat.

The most recent mast seeding occurred in 2018 for beech and in 2019 for oak. Rodent densities were high in 2020 (mean 51.8 individuals per trapping grid; range 19 to 102), low in 2021 (mean 4.4; range 0 to 20), and intermediate in 2022 (mean 14.1; range 1 to 45) (Zwolak et al. 2024a).

At each site, we established a 10 × 10 grid of trapping points, placed 10 m apart and marked with numbered wooden stakes. At each trapping point, we placed 1 wooden live trap (16.5 × 8 × 9.5 cm^3^, produced by “Jerzyk,” Jerzy Chilecki, Białowieża, Poland). Oat flakes and sunflower seeds served as bait, and traps were checked twice daily (morning and evening). Each of the 6 sites was trapped 5 times per year, with each trapping session lasting 4 d. Each site contained 100 traps, checked twice daily (morning and evening). The study was conducted over 3 yr (2020 to 2022), yielding a total of 36,000 trap-nights (6 sites × 5 sessions × 4 nights × 100 traps × 3 years). For logistical reasons, sites were grouped into 3 pairs and pairs were trapped simultaneously.

Captured small mammals were weighed, and their species, sex, and reproductive status recorded. All individuals received unique ear tags (size 1005-1: 2.4 × 9.0 mm^2^, 175 mg, produced by AgnTho's: https://agnthos.se/), and yellow-necked mice additionally received passive integrated transponders (PIT-tags; RF-IDW-2). In 2020, at half of the study sites, adult yellow-necked mice (operationally defined as those weighing 15 g or more) were treated with Frontline Combo Spot-on, a broad-spectrum anti-ectoparasitic applied to the neck. This treatment did not affect subsequent seed foraging patterns (Wróbel et al. 2025).

### Behavioral tests

We assessed the behavioral type of captured individuals using an open-field test to measure activity and exploratory behavior in a novel setting (Montiglio et al. 2012; Bednarz and Zwolak 2022; Celebias et al. 2024). The mouse was released into a 35 × 50 × 30 cm^3^ plexiglass arena partitioned into 4 sections by two 2-cm-high perpendicular barriers. We quantified exploration by counting how many times the mouse crossed the partitions over a 2-min interval. The plexiglass arena was sanitized with 70% ethanol between trials. The tests were recorded using hand-held digital cameras. All testing occurred after the morning trapping session, and individuals were returned to their exact capture locations afterward.

On average, the tested mice participated in 2.0 trials (range: 1 to 11). We did not exclude individuals with a single test because this practice has no benefits and reduces statistical power in the mixed model framework (Martin et al. 2011). Individuals with 1 observation contribute to the estimation of residual variance and fixed effects, even though only those with repeated measures inform the among-individual variance component. The adjusted repeatability of the open-field tests was 0.57 (95% CI: 0.52 to 0.62) on the log-link scale and 0.48 (0.43 to 0.53) on the original scale (Celebias et al. 2024). This metric estimates how consistent an individual's behavior is across multiple trials after accounting for factors such as body mass and sex, and was calculated with Poisson-error family linear mixed models using the *rptR* package (Stoffel et al. 2017). These values are comparable to, or slightly higher than, the average repeatability reported in animal personality studies (0.37 according to a meta-analysis by Bell et al. 2009). We used best linear unbiased predictors as a measure of individual's average value for exploration tendency (Dingemanse et al. 2020). All field procedures were approved by The Local Ethical Committee in Poznań (Approvals No. 24/2018 and 19/2020).

### Foraging trials

To avoid the confounding influence of naturally occurring acorn availability, the foraging experiments were conducted from June to mid-September, before the natural autumnal acorn fall. Beech—the only other significant seed source for mice in these forests—also produces seeds exclusively in autumn. The foraging experiments were carried out at the same sites as the small mammal trapping and immediately followed each trapping session. As with trapping, the sites were paired (3 pairs total), and seed tracking occurred in five 4-d intervals, spaced 3 wk apart, simultaneously in both sites of each pair.

We obtained fully matured acorns, collected the previous autumn, from a forest nursery. For other studies that involved tracking of removed acorns (Celebias et al. 2024; Zwolak et al. 2024a; Wróbel et al. 2025), we marked these acorns with a red plastic tag (20 × 40 mm^2^) secured by a steel wire (100 to 150 mm long, 0.20 to 0.25 mm in diameter). This tagging system weighed about 0.14 g in total.

At about 7 PM at each site, we deployed 4 clusters of 5 acorns (each cluster at least 30 m apart) and checked their status in the morning, starting at 8 AM. The seed clusters were positioned to minimize microhabitat differences, specifically next to tree trunks in the sparse understory. Each cluster was placed inside a Petri dish within the detection loop of a PIT-tag reader's antenna (HPR Plus Handheld PIT Tag Reader, Biomark: https://www.biomark.com/, or Priority1 Design: https://www.priority1design.com.au/) and monitored by a camera trap (Reconyx HyperFire PC800 Professional) mounted 1 m above the ground. This setup recorded the timing of seed removal, allowed the identification of the removing species, and registered identification numbers of mice marked with PIT-tags (Celebias et al. 2024).

In principle, this arrangement could yield up to 2,400 acorns per year (5 acorns × 4 clusters × 6 sites × 4 nights × 5 sessions). In practice, the total was slightly lower because we had to skip several tracking nights in 2021 and 2022 due to unforeseen circumstances. Overall, we offered 7,054 acorns.

### Statistical analyses

All analyses were conducted in R version 4.2.0 (R Core Team 2018). Relative moonlight illumination was calculated using the R package “moonlit” (Śmielak 2023). This measure represents the proportion of moonlight intensity compared with a standardized maximum (set at 1.0), corresponding to the illumination produced by a full moon at zenith at the average Earth–Moon distance. The model incorporates the brightness of the lunar disk, distance to the moon, its position in the sky, atmospheric light propagation, and geographic latitude. We calculated relative moonlight illumination for every 30-min period of the night, as well as the mean relative moonlight intensity per night. Cloud cover data (hourly and per-night averages, on a scale from 0 to 8, where 0 indicates clear sky and 8 represents full cloud cover; converted to percentages on figures) were obtained from the Polish Institute of Meteorology and Water Management—National Research Institute. Rainfall, which often increases small mammal activity (Maestri and Marinho 2014; Wróbel and Bogdziewicz 2015), was extremely rare during our study and therefore omitted from the analysis.

We fitted 4 mixed-effect models, using “glmmTMB” (version 1.1.3; Brooks et al. 2017) and “coxme” (version 2.2-22; Therneau 2024). First, we analyzed how seed removal varied across entire nights to identify conditions under which foraging intensity (per night) was highest. We used a binomial model (logit link function), with seed removal as response variable. The unit of observation was a single seed, coded 1 if removed and 0 otherwise. Predictors were mean moonlight intensity, mean cloud cover, mouse abundance (number of individuals captured during a corresponding trapping session at a given site), and all their interactions (including the 3-way term). Year (2020 to 2022) was not included to avoid confounding effects, as years drastically differed in mouse abundance. Because moonlight and cloud cover are constant within a site × night and seeds within a cluster can be correlated, we included random intercepts for site and seed cluster. Night was not modeled as a random effect because our focus was explicitly on differences among nights, which would otherwise be absorbed by that random term (Fletcher et al. 2010).

Second, we analyzed how mice allocate feeding effort in response to changing moonlight throughout the night. We used a time-to-event (survival) framework in which each seed was treated as an individual subject, and removal was considered the “failure” event. Seeds not removed were right-censored. We subdivided nights into 30-min intervals, assigning each interval moonlight intensity and cloud cover. We also included mouse abundance (individuals per grid per trapping session) to capture density-dependent changes in moonlight preferences. We then fitted a Cox mixed-effects (frailty) model with random intercepts for night and seed cluster (the variance component of site was estimated near zero and model fit did not improve; we therefore omitted this term in the final model).

Third, we analyzed how traits of individual mice influenced their use of different moonlight conditions during foraging. Here, we used a Gamma error family with a log-link function. The response variable was the mean nightly moonlight under which a seed was removed. Predictors included the sex, body mass, and exploration tendency of the mouse that removed the seed. We also included mean nightly cloud cover and its 2-way interactions with individual traits. This approach addresses the question: “Given that a seed was removed, do mouse traits influence the (night-level) conditions under which foraging occurred.” Random intercepts were included for individual mouse, seed cluster, and site.

Finally, we repeated a mouse trait-based analysis for within-night foraging decisions. We used a Tweedie error family with a log-link function. The response variable was the moonlight level at the time of seed removal. The explanatory variables were the same as in the third analysis (sex, body mass, exploration, and their interactions with hourly cloud cover), but we also included night as a random intercept, along with individual mice and seed clusters, as site-level effects remained negligible. Trait analyses were necessarily restricted to removed seeds because unremoved seeds cannot be assigned to individual mice. Consequently, the third and fourth models quantified patterns of use (how removal conditions differed among individuals) rather than selection per se (a comparison between used and unused conditions).

We assessed multicollinearity among predictors and evaluated model diagnostic with “DHARMa” (version 0.4.5; Hartig 2022) and performance packages (version 0.10.9; Lüdecke et al. 2021).

## Results

### Variation in moonlight preferences between nights

**Figure 1:**
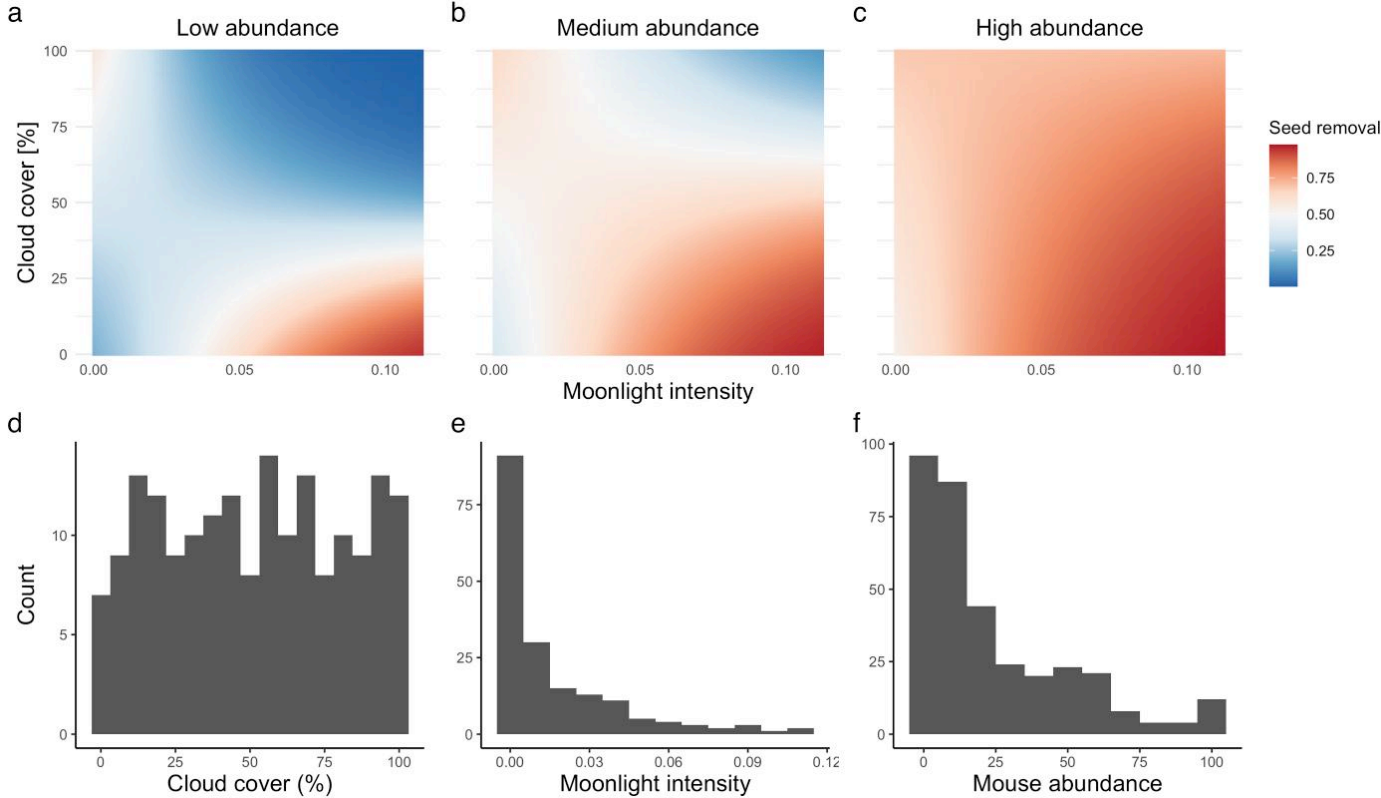
Between-night variation in seed removal as a function of average nightly moonlight intensity, average nightly cloud cover, and the abundance of yellow-necked mice (*A. flavicollis*). (a–c) Model-predicted seed removal across gradients of relative moonlight intensity and cloud cover at 3 levels of mouse abundance: a) low (5 individuals per 1-ha trapping grid; first quartile), b) medium (24 individuals/ha; mean), and c) high (39 individuals/ha; third quartile). Estimates are based on a binomial generalized linear mixed-effects model. (d–f) The observed distributions of d) average nightly cloud cover, e) relative moonlight intensity (nightly averages), and f) mouse abundance (individuals per trapping grid).

Mice removed 42.5% of offered acorns. Seed removal was more intense during nights with high average moonlight intensity (main moonlight effect in Table 1) and this pattern became weaker under high cloud cover (moonlight × clouds interaction in Table 1). However, mouse abundance was the strongest and most consistent driver of seed removal (Table 1). It also modified the influence of the other variables, producing a significant 3-way interaction among average moonlight, average cloud cover, and mouse abundance (Table 1). When mouse abundance was low, seed removal was most intense during nights with high moonlight intensity and low cloud cover, but as mouse abundance increased, foraging occurred under a broader range of cloud conditions, and high removal occurred even under heavier cloud cover (Fig. 1). The range of moonlight conditions in which foraging occurred expanded only when mouse abundance was very high, but even then, foraging remained lower on nights with low average moonlight (Fig. 1c). A secondary, smaller peak in mouse foraging preferences corresponded to dark conditions, when moonlight intensity was low, and cloud cover was high (Fig. 1a and b). However, these conditions corresponded to the majority of nights (Fig. 1e).

**Table 1:** Factors affecting between-night variation in seed removal by yellow-necked mice (*A. flavicollis*).

Variable	Estimate (SE)	Z-value	*P*-value
**Intercept**	−0.707 (0.191)	−3.71	**<0**.**001**
**Moonlight**	0.143 (0.046)	3.07	**0**.**002**
**Clouds**	0.054 (0.039)	1.37	0.171
**Abundance**	1.006 (0.049)	20.47	**<0**.**001**
**Moonlight × clouds**	−0.408 (0.055)	−7.41	**<0**.**001**
**Moonlight × abundance**	0.428 (0.061)	7.07	**<0**.**001**
**Clouds × abundance**	−0.012 (0.041)	−0.29	0.776
**Moonlight × clouds × abundance**	0.314 (0.067)	4.70	**<0**.**001**

Estimates are based on a binomial generalized linear mixed model and represent change in the outcome for a 1-standard-deviation increase in the predictor. “Moonlight” and “Clouds” are per-night mean values, while “Abundance” reflects the density of conspecific mice during a given 4-d trapping session on a given trapping grid. All predictors have been zero-centered and standardized. Significant *P*-values are presented in bold. *N* = 7,054 seeds deployed across 190 nights.

### Variation in moonlight within nights

**Figure 2:**
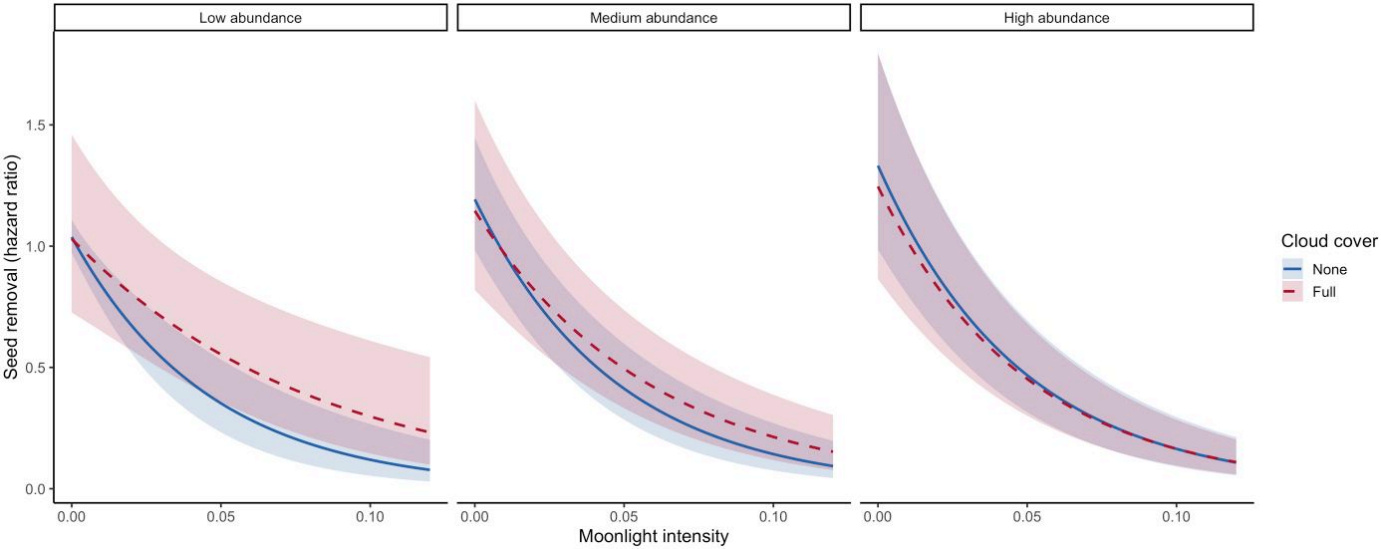
Within-night variation in seed removal as a function of relative moonlight intensity, cloud cover (none: 0%; full: 100%), and the abundance of yellow-necked mice (*A. flavicollis*): low, 5 captured individuals per 1-ha trapping grid (first quartile); medium, 24 individuals/grid (mean); and high, 39 individuals/grid (third quartile). Estimates based on a mixed-effects Cox model.

Mice strongly avoided night periods when moonlight was bright (main moonlight effect in Table 2). Cloud cover reduced this avoidance, but only when mouse abundance was low; this moderating influence was lost when mouse abundance was high (moonlight × clouds × abundance interaction in Table 2; Fig. 2).

**Table 2:** Factors affecting within-night variation in seed removal by yellow-necked mice (*A. flavicollis*).

Variable	Estimate (SE)	Odds ratio	Z-value	*P*-value
**Moonlight**	−0.634 (0.059)	0.53	−10.76	**<0**.**001**
**Clouds**	0.011 (0.047)	1.01	0.23	0.821
**Abundance**	0.134 (0.085)	1.14	1.56	0.118
**Moonlight × clouds**	0.057 (0.061)	1.06	0.94	0.345
**Moonlight × abundance**	−0.088 (0.041)	0.92	−2.15	**0**.**031**
**Clouds × abundance**	−0.067 (0.040)	0.94	−1.66	0.096
**Moonlight × clouds × abundance**	−0.094 (0.042)	0.91	−2.24	**0**.**025**

Estimates are from a mixed-effects Cox model and represent the change in seed removal for a 1-standard-deviation increase in each predictor. The hazard ratios (“Odds ratio” column) show how each predictor modifies the instantaneous risk (hazard) of seed removal within a night. Relative moonlight intensity was estimated in 30-min intervals, and cloud cover measured in hourly intervals; both were matched to the corresponding seed-removal events. All predictors have been zero-centered and standardized. Significant *P*-values are presented in bold. *N* = 2,486 removal events, 89,294 “failures” to remove.

### Individual traits and between-night variation in foraging

Males and lighter mice foraged more often during nights with lower average moonlight level (Table 3), while exploration tendency did not predict moonlight preferences. The effects of sex, body mass, and exploration tendency on foraging activity were unaffected by cloud cover (non-significant main and interaction effects of Clouds in Table 3).

**Table 3:** Effects of individual traits of yellow-necked mice (*A. flavicolis*) and cloud cover on between-night moonlight preferences during foraging.

Variable	Estimate (SE)	Z-value	*P*-value
**Intercept**	−5.717 (0.345)	−16.55	**<0**.**001**
**Body mass**	0.337 (0.155)	2.18	**0**.**029**
**Sex (M)**	−0.762 (0.344)	−2.22	**0**.**027**
**Exploration**	−0.090 (0.614)	−0.15	0.884
**Clouds**	0.308 (0.164)	1.88	0.060
**Body mass × clouds**	−0.044 (0.100)	−0.44	0.659
**Sex (M) × clouds**	−0.256 (0.201)	−1.27	0.203
**Exploration × clouds**	0.173 (0.321)	0.54	0.591

“Sex” denotes preferences of males (M) relatively to females. “Clouds” are per-night mean cloud cover values. Estimates are from a generalized linear mixed-effects model with a Gamma error distribution and a log-link function. All predictors have been zero-centered and standardized. Significant *P*-values are presented in bold. *N* = 1,230 seeds removed by 209 individuals.

### Individual traits and within-night variation in foraging

**Figure 3:**
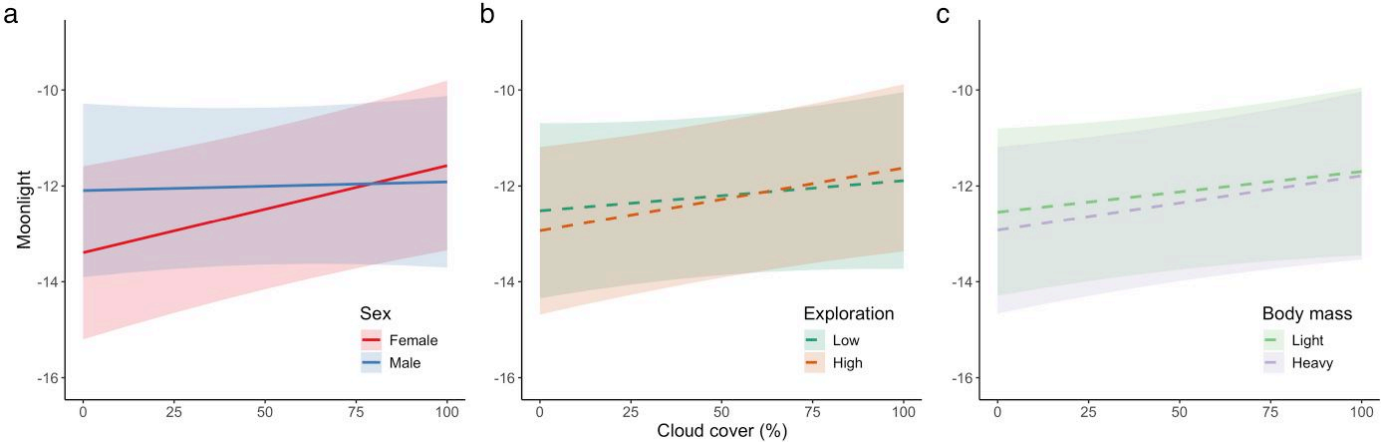
Effects of individual traits on within-night moonlight preferences in foraging yellow-necked mice (*A. flavicolis*). Panels represent the influence of a) sex, b) exploration tendency (“Low”: first quartile; “High”: third quartile), and c) body mass (“Light”, first quartile: 25.0 g, “Heavy”, third quartile: 35.0 g) on relative moonlight intensity (*y*-axis, log scale) during seed-removal events, across varying levels of cloud cover (*x*-axis). Lines represent estimates from generalized mixed-effects models (solid for significant interaction effects; dashed for non-significant), and shaded areas indicate 95% intervals.

Females foraged under lower moonlight intensity than males, but this difference disappeared with increasing cloud cover (Sex and Sex × Clouds effects in Table 4; Fig. 3a). Exploration tendency and body mass did not predict moonlight preferences (non-significant main and interaction effects in Table 4; Fig. 3b, c).

**Table 4:** Effects of individual traits of yellow-necked mice (*A. flavicolis*) and cloud cover on within-night moonlight preferences during foraging.

Variable	Estimate (SE)	Z-value	*P*-value
**Intercept**	−12.311 (0.853)	−14.44	**<0**.**001**
**Body mass**	−0.153 (0.155)	−0.99	0.324
**Sex (males)**	0.322 (0.350)	0.92	0.356
**Exploration tendency**	−0.022 (0.621)	−0.04	0.972
**Clouds**	0.634 (0.226)	2.81	**0**.**005**
**Body mass × clouds**	0.075 (0.102)	0.74	0.459
**Sex (males)×clouds**	−0.570 (0.252)	−2.26	**0**.**024**
**Exploration × clouds**	0.449 (0.475)	0.95	0.345

“Sex” denotes preferences of males (M) relatively to females. “Clouds” correspond to cloud cover at the time of seed removal. Estimates are from a generalized linear mixed-effects model with a Tweedie error distribution and a log-link function. All continuous predictors have been zero-centered and standardized. Significant *P*-values are presented in bold. *N* = 1,012 acorns removed by 185 individuals.

## Discussion

We found that yellow-necked mice exhibit marked foraging responses to moonlight, even in closed-canopy broadleaf forests where its influence might be expected to be weak. Notably, these responses differed depending on the temporal scale of analysis: the conditions under which seed removal was highest when averaged across entire nights were not the same as those that promoted seed removal during specific periods within nights. Foraging patterns were further shaped by conspecific abundance, and, to a lesser extent, by individual traits. Two main insights emerge that can affect how we interpret moonlight-preference studies: (i) the influence of moonlight depends on the temporal scale of analysis, and (ii) its strength declines with higher conspecific density. Although individual traits also play a role, their effects appear less consistent.

The finding that mice foraged more intensely during bright nights is consistent with the visual acuity hypothesis, which states that bright conditions improve foraging efficiency (Prugh and Golden 2014) and may also enhance the ability to detect approaching predators (Traill et al. 2016; Hart et al. 2020). We initially predicted the opposite pattern, described by the predation risk hypothesis, because rodents in temperate forests face intense predation pressure (Jędrzejewski and Jędrzejewska 1993), and at least some predators increase their activity with moonlight (Wereszczuk and Zalewski 2023). In principle, this should lead to increased vigilance and reduced foraging during bright conditions (Abramsky et al. 2002). Moreover, non-visual foragers such as mice typically avoid moonlight (Prugh and Golden 2014), though many exceptions are documented (Longland and Price 1991; Prugh and Brashares 2010; Maestri and Marinho 2014; Berry and Stapp 2024). Some of these exceptions may relate to microhabitat use: in certain systems, rodents that forage during full moon do so mainly under dense vegetation (Guiden et al. 2023; Berry and Stapp 2024). However, managed broadleaf forest at our study sites has quite homogenous ground cover, and we placed seed clusters specifically to minimize potential habitat differences (see “Methods”) and reduce potential shifts in foraging microhabitats.

By contrast, within-night foraging patterns supported the predation risk hypothesis: mice concentrated their activity during darker intervals (low moon brightness and high cloud cover). This suggests that foraging activity reflects processes operating at 2 temporal scales, with different conditions shaping activity across nights versus within nights. We speculate that mice benefit from having sufficient light to forage effectively but must also minimize the risk of being spotted by predators, thus they allocate foraging effort to darker intervals even on otherwise bright nights. Consequently, this leads to seemingly contradictory patterns when comparing whole-night versus within-night scales.

In agreement with our predictions, intraspecific density shaped both across-nights and within-night foraging preferences. Across nights, mice became less selective and intensified foraging under a wider spectrum of moonlight intensity and—particularly—cloud cover. Within nights, conspecific abundance did not alter the response to moonlight levels, but reduced sensitivity to cloud cover. However, the influence of abundance on within-night foraging, while evident, was less dramatic than at the across-nights scale. This likely reflects the greater variation in mouse abundance among nights, driven by pronounced fluctuations over months and years, compared with within-night variation, which mostly represented moderate differences across sites.

These findings show that temporal preferences can be just as sensitive to conspecific density as spatial preferences, though the latter have traditionally received more attention. Two potential mechanisms may explain the abundance-driven decline in selectiveness. Classical theory, originally developed for habitat selection but just as relevant to temporal selection, stresses the dominant role of intraspecific competition (Fretwell and Lucas 1970; Pulliam 1988). As density increases and more individuals forage in a high-quality patch or period, competition costs rise until foraging in lower-quality options becomes equally profitable. Alternatively, the “dilution effect” could also play a role: predation risk may decrease on a per-capita basis as forager populations increase (Finkbeiner et al. 2012; Gorosito et al. 2024). Both mechanisms have been found in rodents, including yellow-necked mice, in the context of habitat selection (Morris 1996; Zwolak et al. 2016, 2018).

Individual traits also mattered, but their influence differed at the between-night and within-night scales. The between-night analysis averages conditions over entire nights, perhaps capturing broad foraging decisions. Traits like sex and body mass emerged more strongly at this scale: males and lighter individuals foraged more during darker nights. Unexpectedly, these patterns remained unchanged by cloud cover, possibly because nightly averages obscured variation in cloud conditions.

In contrast, the within-night analysis focused on finer time windows that captured immediate responses to moonlight, cloud cover, and their joint effects. Females were more sensitive to cloud cover than males; under clear skies, females foraged at lower moonlight intensities than males, but this sex difference diminished as cloud cover increased. This pattern suggests more cautious behavior by females and is consistent with laboratory studies on risk-taking in laboratory rodents (Jolles et al. 2015; Zambetti et al. 2019). Contrary to our predictions, exploration in the open-field test was not linked to mouse responses to moonlight. While unexpected, this aligns with emerging studies indicating a more complex relationship between behavioral types, risk-taking, and survival (Moiron et al. 2020; Laskowski et al. 2022). Overall, these results offer mixed support for the hypothesized roles of individual traits, indicating that the relevance of sex and body mass may depend on the temporal scale of analysis, while exploration tendencies do not appear to shape moonlight preferences of foraging animals.

Although moonlight and cloud cover jointly determine light availability, we treated them as separate predictors because their effects may extend beyond illumination. Our hypotheses were primarily framed around light conditions, but cloud cover can also influence behavior through weather-related mechanisms such as changes in temperature, humidity, or precipitation risk. Consistent with this view, our results show that cloud cover sometimes modulated the effects of moonlight, but also had independent effects, indicating that both light- and weather-related pathways may shape rodent foraging decisions.

In summary, our findings indicate that moonlight still plays a significant role even in dense forests (Bischof et al. 2024) and help reconcile conflicting results from previous work. Most studies found that nocturnal rodents avoid moonlight (Kotler 1984; Price et al. 1984; Bowers 1988; Viviano et al. 2022; Taylor et al. 2023; Bischof et al. 2024; also meta-analysis: Prugh and Golden 2014), but other found preference (Longland and Price 1991; Prugh and Brashares 2010; Maestri and Marinho 2014; Berry and Stapp 2024), and yet other indifference (Moll et al. 2020). We demonstrated that individual traits shape the patterns of moonlight preference, and conspecific abundance blunts them. Importantly, the factors that influence which night mice choose to forage differ from those that determine how they time their foraging bouts within a night. Therefore, relying solely on nightly averages of light conditions risks overlooking important within-night behaviors.

## Data Availability

Analyses reported in this article can be reproduced using the data provided by Zwolak et al (2026).
